# Profiling the Effect of Targeting Wild Isocitrate Dehydrogenase 1 (IDH1) on the Cellular Metabolome of Leukemic Cells

**DOI:** 10.3390/ijms23126653

**Published:** 2022-06-15

**Authors:** Mohammed Razeeth Shait Mohammed, Faisal Alzahrani, Salman Hosawi, Hani Choudhry, Mohammad Imran Khan

**Affiliations:** 1 Department of Biochemistry, Faculty of Science, King Abdulaziz University, Jeddah 21589, Saudi Arabia; faahalzahrani@kau.edu.sa (F.A.); shosawi@kau.edu.sa (S.H.); hchoudhry@kau.edu.sa (H.C.); 2 Centre of Artificial Intelligence for Precision Medicines, King Abdulaziz University, Jeddah 21589, Saudi Arabia; 3 Embryonic Stem Cells Unit, King Fahd Medical Research Center, King Abdulaziz University, Jeddah 21589, Saudi Arabia

**Keywords:** wild-type IDH1, metabolomics, glutamine metabolism, reactive oxygen species, OXPHOS

## Abstract

Leukemia is one of the most common primary malignancies of the hematologic system in both children and adults and remains a largely incurable or relapsing disease. The elucidation of disease subtypes based on mutational profiling has not improved clinical outcomes. IDH1/2 are critical enzymes of the TCA cycle that produces α-ketoglutarate (αKG). However, their mutated version is well reported in various cancer types, including leukemia, which produces D-2 hydroxyglutarate (D-2HG), an oncometabolite. Recently, some studies have shown that wild-type IDH1 is highly expressed in non-small cell lung carcinoma (NSCLC), primary glioblastomas (GBM), and several hematological malignancies and is correlated with disease progression. This work shows that the treatment of wild-type IDH1 leukemia cells with a specific IDH1 inhibitor shifted leukemic cells toward glycolysis from the oxidative phosphorylation (OXPHOS) phenotype. We also noticed a reduction in αKG in treated cells, possibly suggesting the inhibition of IDH1 enzymatic activity. Furthermore, we found that IDH1 inhibition reduced the metabolites related to one-carbon metabolism, which is essential for maintaining global methylation in leukemic cells. Finally, we observed that metabolic alteration in IDH1 inhibitor-treated leukemic cells promoted reactive oxygen species (ROS) formation and the loss of mitochondrial membrane potential, leading to apoptosis in leukemic cells. We showed that targeting wild-type IDH1 leukemic cells promotes metabolic alterations that can be exploited for combination therapies for a better outcome.

## 1. Introduction

Leukemia is a leading cause of mortality worldwide [1]. The frequency of leukemia is recognized by mutation, and chromosomal aberration and translocation result in oncogenic activities. The present treatments continue to cause harmful side effects [2,3]. Recently, a new small molecular targeted inhibitor was designed to transform deadly diseases into controllable conditions [4]. The impact of *IDH* mutations on AML prognosis remains somewhat debatable, although generally, a minor outcome is seen with *IDH1* mutations, and a relatively favorable prognosis may be seen with *IDH2* mutations. Among these new molecular targets, isocitrate dehydrogenase family (IDH1/2) enzymes have emerged as desirable targets.

IDH1/2 family enzymes are metabolic enzymes that catalyze the oxidation of isocitrate to alpha-ketoglutarate in the citric acid cycle and produce the crucial reducing equivalent NADPH [5]. Numerous new emerging reports of exome sequencing have identified frequent mutations in the IDH1 enzyme or its homolog IDH2 in leukemia and other cancers. *IDH1* and 2 are important genes in acute myeloid leukemia [6,7,8].

Recently, it has been shown that wild-type IDH1 is highly expressed in a variety of cancer types, including non-small cell lung carcinoma (NSCLC), primary glioblastomas (GBM), and several hematological malignancies and is correlated with disease progression [9]. Cancer cells are capable of uncontrolled and quick proliferation without the induction of external stimuli. To meet their energy needs, cancer cells must undergo metabolic reprogramming other than Warburg effects. Leukemic cells are associated with high oxidative phosphorylation and show chemoresistance. IDH mutations play a significant role in leukemogenesis and metabolism. IDH mutants downregulate ROS production and metabolic alteration. In leukemic cells, the TCA cycle plays a prominent role in energy metabolism and glutaminolysis. Glutamine is an important metabolite in the regulation of GSH and the maintenance of redox potential.

IDH1 inactivation results in decreased NADPH, with consequent exhaustion of GSH and increased levels of ROS, reduction of lipid biosynthesis, and enhanced histone methylation and differentiation marker expression. Similarly, shRNAs targeting IDH1 decreased the in vitro and in vivo growth of NSCLC cell lines [10]. Furthermore, IDH1 silencing in a diffuse large B cell lymphoma (DLBCL) cell line decreased αKG and GSH production, with a subsequent increase in ROS and tumor growth reduction. These results showed that wild-type IDH1 is an essential therapeutic target in cancer.

Since the wild-type IDH1 enzyme is a crucial metabolic enzyme, in this study, we aimed to understand the metabolic impact of targeting wild-type IDH1 using GSK864 (an IDH1 mutant inhibitor that also inhibits wild-type IDH1 at high concentrations) [11] in leukemia cell types as an experimental model. The results showed that targeting wild-type IDH1 inhibition shifted leukemia cell metabolism toward oxidative phosphorylation from glycolysis, enhanced ROS, and deregulated membrane potential, leading to cell death.

## 2. Results

### 2.1. Wild-Type IDH1 Inhibition Reduces Leukemic Cell Proliferation

**Figure 1:**
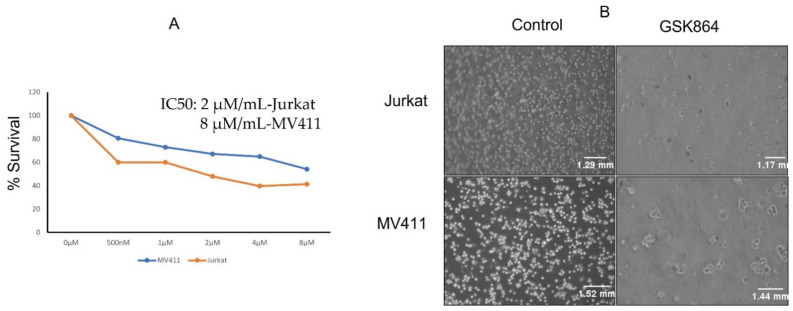
GSK864 treatments reduced cell proliferation and altered cellular morphology (**A**,**B**). MTT assays were performed to determine cell viability upon treatment with different concentrations of GSK864 for 48 h in both Jurkat and MV4-11 cells. All the images were captured using a Nikon phase contrast microscope at 20×. *p* < 0.01.

The leukemic cell lines Jurkat and MV4-11 were treated with a wild-type IDH1 inhibitor, namely GSK864, and their cell proliferation assay was examined (Figure 1A). The IC50 values of GSK864 for these cell lines were examined after 48 h of treatment. Briefly, GSK864 treatment at a concentration of 2 μmol/mL showed significant inhibitory effects on the proliferation of both the Jurkat and MV4-11 cell lines. Physiological examination under a microscope also showed that the number of cells increased toward apoptotic morphology (chromatin condensation) (Figure 1B).

### 2.2. Wild-Type IDH1 Inhibition Alters Global Leukemic Cells’ Metabolic Landscape

**Figure 2:**
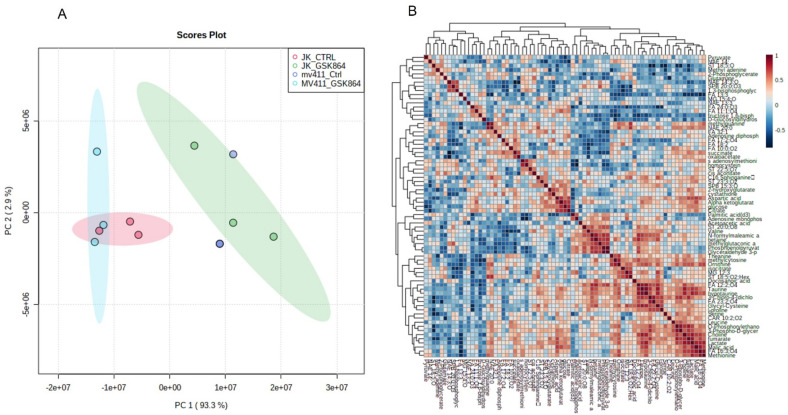

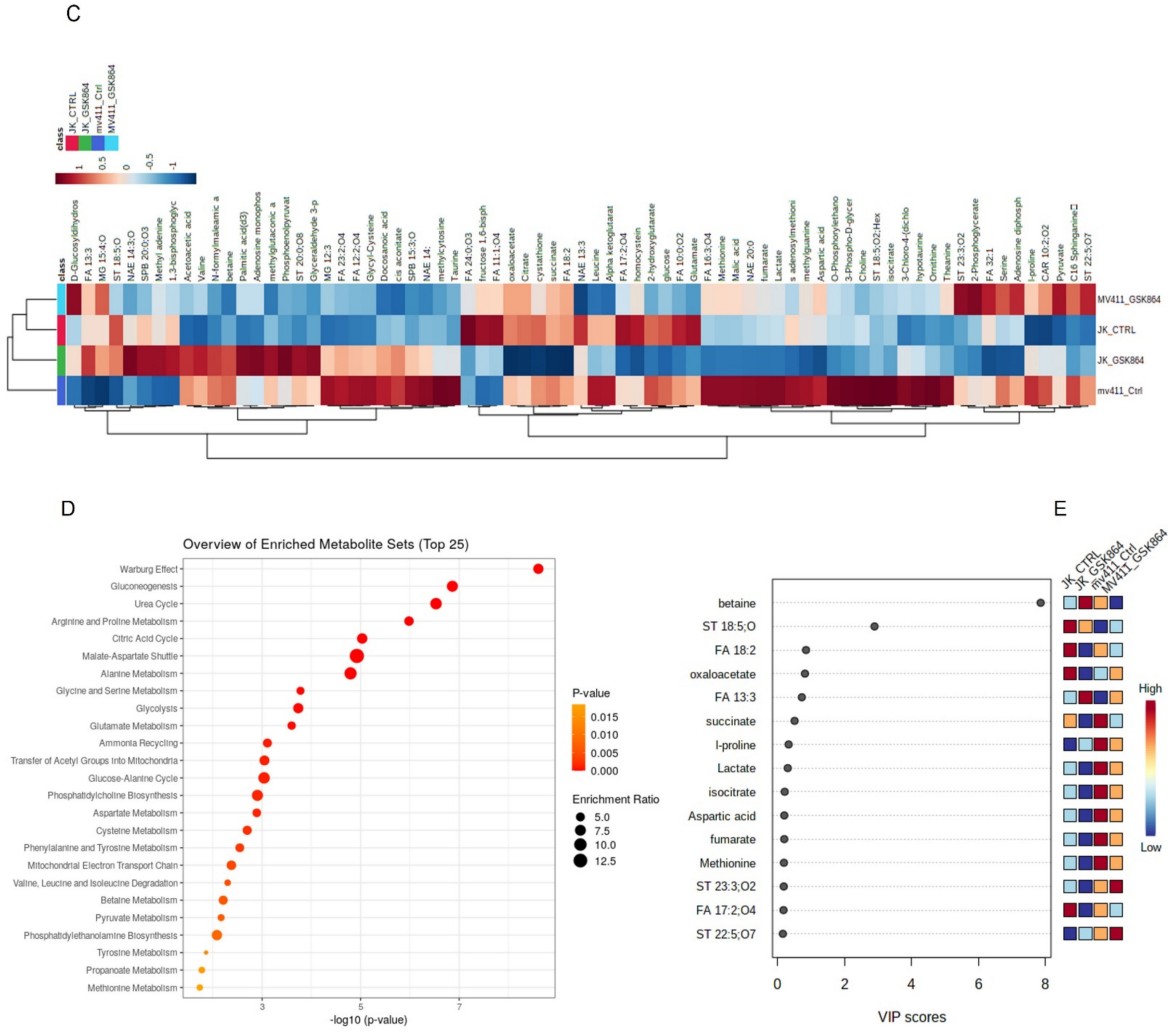
Metabolomic analysis of leukemic cells treated with IDHi: (**A**) PCA analysis of total metabolites of Jurkat and MV4−11cells; (**B**,**C**) correlation and expression heatmap of differential metabolites expressed in control and GSK864-treated cells; (**C**) top pathways enriched in Jurkat and MV4-11 control and GSK864-treated cells; (**D**) The enriched metabolic pathways altered by IDHi (**E**) VIP score for differentially expressed metabolites during GSK864 treatment.

To investigate the metabolic landscape of leukemic cells treated with wild-type IDH1 inhibitor, i.e., GSK864, metabolites were extracted from untreated (mock) and GSK864-treated Jurkat and MV4-11 cells. Untargeted metabolomics was performed using our house facility of GC–MS/MS. The spectra of the three biological replicates from the cell lines (Jurkat and MV4-11) were obtained. The intracellular metabolites’ GC–MS/MS spectral separation (TIC, total ion chromatogram) is shown in Supplementary Figure S1. A broadband comprehensive metabolite list with identification, *p*-value, features, and peak intensity is shown in Table S1. HMDB databases were used to identify the metabolic markers. The metabolomic variation between each sample was demonstrated by multivariate analysis using a PLS-DA score plot (Figure 2A), with FDR-corrected values of *p* < 0.05 and *q* < 0.05. A comparative representation of the identified metabolites between the untreated and treated samples was created using a metabolic heat map with an FDR-corrected q-value of <0.05, which was established by ward clustering (Figure 2B,C). Results GSK864 (IDHi) treatment.

For the pathway enrichment analysis, the differential regulated metabolites were mapped to the KEGG database using MetaboAnalyst 5.0. The top 25 enriched pathways were identified (Figure 2D), with significance defined as *p* < 0.05 (Supplementary Table S2). The enriched pathways involved were the Warburg effect, gluconeogenesis, urea cycle, malate–aspartate shuttle, glycolysis, glycine and serine metabolism, and glutamate metabolism. The top significantly regulated metabolites are shown in Figure 2E.

### 2.3. Wild-Type IDH1 Inhibition Modulates Warburg Phenotype in Leukemic Cells

**Figure 3:**
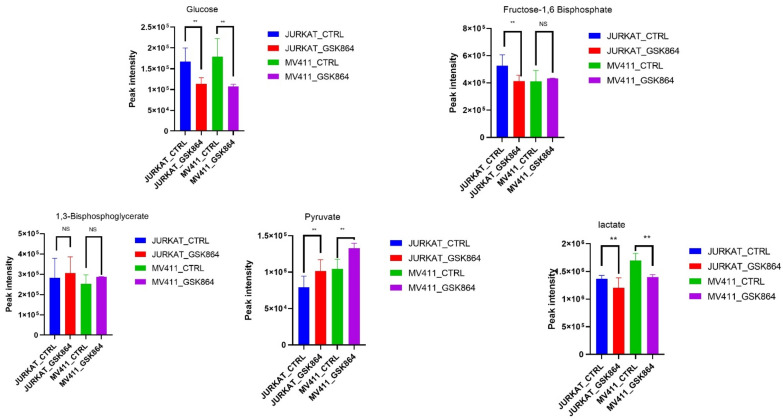
IDHi altered crucial cellular energy pathways in leukemic cells. Quantitative levels of various metabolites involved in glycolysis pathways of control and GSK864-treated cells; ** *p* < 0.00.

We aimed to uncover the Warburg effect metabolic characteristics underlying the increased forming ability in IDHi. Most cancer cells utilize a large amount of glucose and produce lactic acid as a fuel source for energy production. Remarkably, IDHi displayed enrichment of metabolites related to glycolytic intermediates, including fructose-1,6-bisphosphate (F1,6P) and 1,3-bisphosphoglycerate (3PG), compared with untreated leukemic cells (Figure 3). In addition, the metabolites of another glucose-utilizing pathway, monophosphate (AMP), were significantly accumulated in IDHi (Figure 3). Consistent with an increase in glycolysis, it was enhanced in IDHi compared with untreated leukemic cells. These identified metabolic profiles were under anaerobic glycolysis, which impacted the Warburg effect—a well-known hallmark of rapidly proliferating mammalian cells.

**Figure 4:**
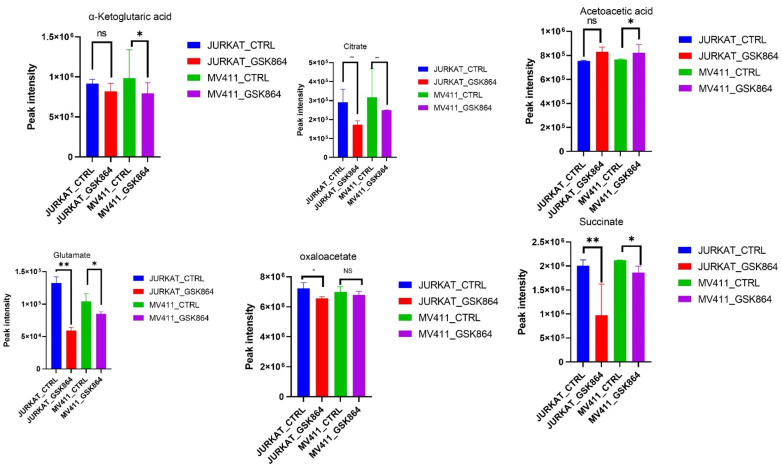
IDHi altered crucial TCA cycle pathways in leukemic cells. Quantitative levels of various metabolites involved in TCA cycle pathways of control and GSK864-treated cells, * *p* < 0.01. ** *p* < 0.00.

IDHi displayed enrichment of metabolites related to the TCA cycle, OXPHOS intermediates. However, some TCA cycle metabolites, including fumarate, citrate, α-ketoglutarate, acetoacetate, glutamate, oxaloacetate, and pyruvate, were reduced in IDHi (Figure 4). These results suggest that the leukemic cells switched to a more glycolytic phenotype from an oxidative phosphorylation (OXPHOS) phenotype and were regulated by IDHi (Figure 4).

### 2.4. Wild-Type IDH1 Regulates One-Carbon Metabolism in Leukemic Cells

**Figure 5:**
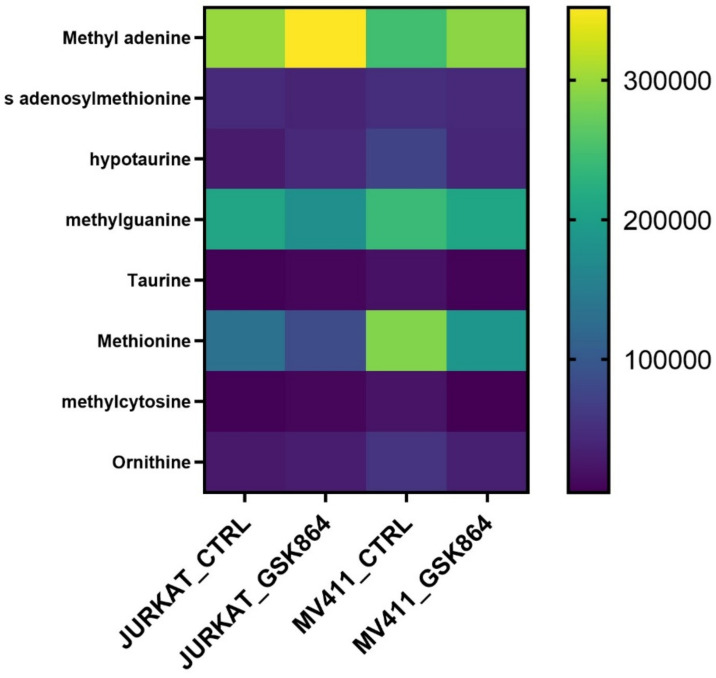
IDHi alters one-carbon metabolism in leukemic cells. Quantitative levels of various metabolites involved in control and GSK864-treated cells involved in one-carbon metabolism.

Methionine, taurine, and hypotaurine are essential in one-carbon metabolism since methionine is chronologically converted into SAM (S-adenosylmethionine). The methyl group uses it after modification of histone, RNA, and DNA methylation. Taurine and hypotaurine are cysteine derivatives and crucial metabolites implicated in one-carbon metabolism. The results show that IDHi activates metabolites involved in one-carbon metabolism, such as methionine, taurine, SAM, methyl cytosine, and ornithine levels (Figure 5). This result indicates that IDHi alters the methylation shape in leukemic cells.

### 2.5. Wild-Type IDH1 Inhibition Promotes Mitochondrial Depolarization in Leukemic Cell

**Figure 6:**
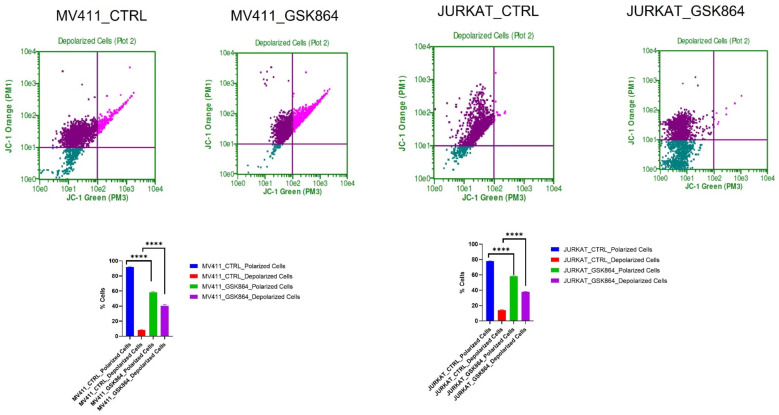
IDHi alters MMP in leukemic cells. Quantitative levels of mitochondrial membrane potential alteration in control-treated leukemic cell; **** *p* < 0.0000.

The IDHi in leukemic cells activates TCA cycle intermediatory metabolites in the mitochondria, resulting in respiration inhibition and inducing mitochondrial depolarization. IDHi reduced the polarization of the mitochondrial membrane in MV4-11 and Jurkat cells, as evidenced by JC-1 dye (Figure 6). The results showed an approximately 15% reduction in mitochondrial polarization and a 17% increase in mitochondrial depolarization in MV4-11 cells treated with GSK864 compared with controls. Still, Jurkat cells showed about a 30% reduction in mitochondrial polarization and a 40% increase in mitochondrial depolarization with IDHi. Both decreases in polarization and increases in depolarization of the mitochondrial membrane potential are characteristic features of mitochondria-mediated apoptosis (Figure 6).

### 2.6. Wild-Type IDH1 Inhibition Promotes the Accumulation of Intracellular ROS and Induces Apoptosis

**Figure 7:**
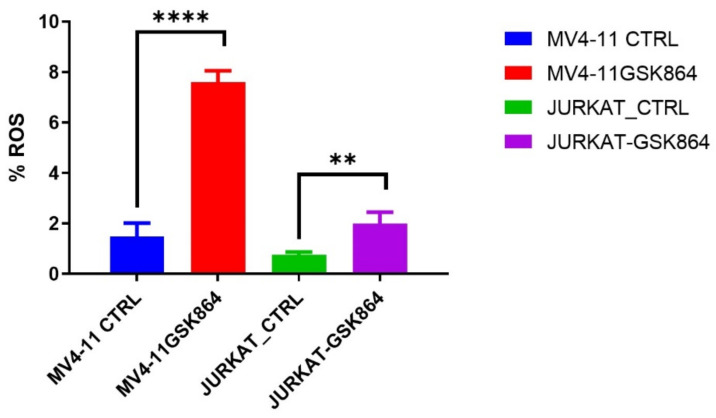
IDHi induces ROS in leukemic cells. Quantitative levels of ROS in control and treated leukemic cells; ** *p* < 0.00, **** *p* < 0.0000.

ROS (reactive oxygen species) are involved in the loss of growth control, genomic instability, and invasiveness. References [12,13] showed that disproportionate ROS is harmful to cells, resulting in oxidative damage to RNA, DNA, and proteins. In leukemic cells, IDHi elevated the ROS level in MV4-11 and Jurkat cells. We observed that IDHi induced intracellular ROS levels of 6% in MV4-11 cells, and Jurkat cells showed a 2% induction in intracellular ROS levels (Figure 7).

**Figure 8:**
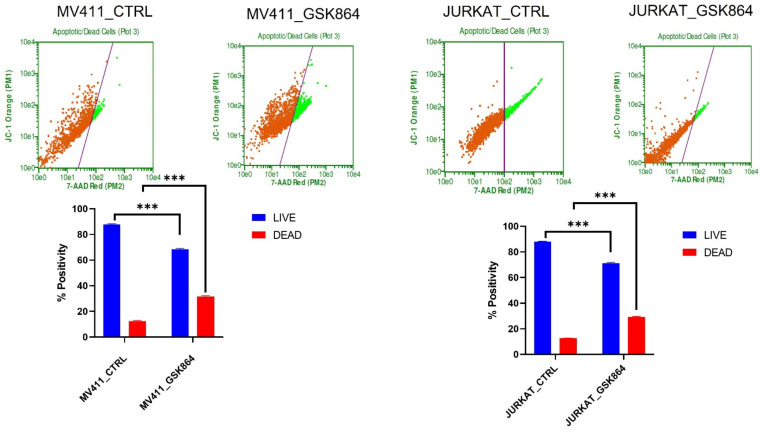
IDHi induced cell death in leukemic cells. The live and the dead assays were performed in control and GSK864-treated leukemic cells; *** *p* < 0.000.

Wild-type IDH1 is a potential therapeutic target; we treated leukemic cells with GSK864. 7-Aminoactinomycin D (7AAD) staining was used to differentiate live cells from apoptotic cells. We observed that IDHi induced 17% of apoptosis in MV411 and Jurkat cells. IDHi prevented cell proliferation in leukemic cells (Figure 8).

## 3. Discussion

In leukemia, the *IDH1*/*IDH2* gene restructures the enzyme for isocitrate while increasing the affinity for α-ketoglutarate (αKG) with the production of 2HG [14]. Under functional conditions, the IDH isoforms produce metabolite products of the Krebs cycle. By contrast, IDH2 (the isoform localized in mitochondria) and IDH1 (the isoform localized in the cytoplasm and peroxisomes) are involved in oxidative decarboxylation, generate αKG from isocitrate, and yield reduced NADPH from NADP+. IDH1/2 mutants catalyze the conversion of α-KG α-ketoglutarate to D2HG-D-2-hydroxyglutarate and promote the glutaminolysis pathway to support the carbon requirement for anaplerosis to fuel the Krebs cycle [14]. In addition, they constrain αKG synthesis by reducing glycolytic influx and diminishing the Krebs cycle [15]. Most of the observed IDH1 mutations result in an amino acid substitution at R132 in the enzyme. Mutational IDH1 induces the neomorphic activity of the enzyme that changes the product from alpha-ketoglutarate (αKG) to D-2-hydroxyglutarate (D-2HG), which accumulates to extremely high levels in tumors with IDH1 mutations (~100-fold increase) [6]. D-2 HG inhibits αKG-dependent dioxygenase enzyme activity, including Jumonji C domain-containing histone demethylases and Tet 5-methylcytosine (5 mC) hydroxylases, resulting in epigenetic alterations and perturbed cellular differentiation that may contribute to tumorigenesis [4].

First, we explored the global metabolic landscape of both cell lines treated with IDHi using untargeted metabolomics. Furthermore, using this approach, we identified novel metabolites and critical regulatory pathways affected by IDHi in leukemic cells. The data showed that IDHi resulted in the modification of the overall cellular metabolic rate of leukemic cells. The most perilous pathways regulated by IDHi are the Warburg effect, glycolysis, TCA cycle, one-carbon metabolism (glycine–serine metabolism), and lipid metabolism.

We observed that IDHi in leukemic cells shifted cell metabolism toward a glycolytic phenotype, as suggested by the increased pyruvate levels and decreased lactate levels. This metabolic adaptation is well known to play a substantial role in energy metabolism. Glutamate–glutamine metabolism promotes glutathione biosynthesis [16]. A significant part of glutamine metabolism is the reduction in ROS by increasing antioxidants and switching energy production from glutamine via the TCA cycle in cancer cells [17]. Our results showed that IDHi reduced glutamate levels and thereby limited intracellular uptake, resulting in increased oxidative stress and OXPHOS in leukemic cells.

The mechanisms by which methylation arises and its effects on cancer are becoming better understood. *IDH1* encodes enzymes that convert isocitrate into α-ketoglutarate, and it is a cofactor for certain DNA demethylases, leading to hypermethylation in genomic CpG histone methylation. IDHi also competitively inhibited the formation of isocitrate into α-ketoglutarate, which inhibits the TCA cycle and metabolites involved in one-carbon metabolism. Our data suggest that IDHi results in reduced methionine and serine metabolism. However, these claims require additional confirmatory experiments. Overall, we showed that targeting wild-type IDH1 in leukemic cells invoked redox-linked stress by modulating pathways associated with the glutamine pathway.

## 4. Materials and Methods

### 4.1. Leukemic Cell Culture

The wild-type IDH1-containing leukemic cell lines Jurkat and MV411 were obtained from ATCC, USA. Leukemic cell lines were cultured and maintained in RPMI (Gibco, Invitrogen, Carlsbad, CA, USA) supplemented with 10% fetal bovine serum (FBS; Gibco one-shot, USA) and 50 U/mL pen strep (Gibco) in 5% CO_2_ at 37 °C. The cells were treated with either vehicle control or GSK864 (Sigma SML1767, St. Louis, MI, USA) for 48 h [18].

### 4.2. MTT Assay

Briefly, 1 × 10^4^ cells were seeded in 96-well plates, and cells were treated with different concentrations of GSK864. After 48 h of treatment, 10 µL of MTT (5 mg/mL) at a ratio of 1:10 was added. Then, the plate was incubated for another three hours at 37 °C in a CO_2_ incubator. After incubation, the medium was removed and 100 µL of DMSO was added, and the solution was incubated at RT for 10 min. The OD was measured at 540 nm [1].

### 4.3. Metabolites Extraction

Metabolites were extracted from cells treated with GSK864. Treated cells were lysed immediately using a tissue homogenizer with a solution of ice-cold methanol: acetonitrile: water at a ratio of 2:1:1 *v*/*v*, vortexed for 30 s, incubated for 24 h at −20 °C, and spun for 15 min at 13,000 rpm at 4 °C [19,20,21]. The supernatant was dried entirely in a speed vac. The derivatization was carried out in two steps. In step 1, protection of carbonyl function was achieved by adding 20 μL of 40 mg/mL solution of methoxyamine hydrochloride in pyridine and heated at 30 °C for 90 min. Step 2 increased the volatility of the small molecules; the samples were treated with a derivatizing agent, 180 μL of N-,ethyl-N-trimethylsilytrifluroroacetamide with 1% trimethylchlorosilane, at 37 °C for 30 min [16].

### 4.4. GC-Mass Spectrometry

An Agilent 7890 GC oven was heated at 8 °C/min from 60 °C (1 min initial time) to 315 °C, resulting in a 31.875 min run time, and was cooled down to 60 °C. A quantity of 1 µL was injected into the Agilent split/splitless injector at 250 °C by a 10 µL syringe with 4 sample pumps, one pre-injection wash, and two post-injection washes using both solvent A and solvent B. The raw .D files were converted into a data format.

### 4.5. Data Processing and Analysis

The raw data were processed using open access to the XCMS online database. Peaks were searched against human metabolites in the Human Metabolome Database. Pathway analysis and statics were performed using Metaboanalyst [19,20,21].

### 4.6. ROS Assay

A total of 1 × 10^6^ cells were plated on six-well plates. The cells were treated with the GSK864 compound for 48 h. The CELLROX (Invitrogen) was used to measure the level of intracellular reactive oxygen species (ROS) in live cells. GSK864-treated cells were incubated in a culture medium with 500 nM CELLROX for 60 min at 37 °C and 5% CO_2_. Flow cytometry immediately analyzed the samples using 488 nm excitation for the CellROX^®^ Green [22,23].

### 4.7. Mitochondria Membrane Potential Assay

A total of 1 × 10^6^ cells were plated on six-well plates. The cells were treated with the GSK864 compound for 48 h, and a A volume of 2 μL 100X JC-1 solution and 2 μL of 7-AAD was then added to each well. Plates were incubated for 30 min at 37 °C in a 5% CO_2_ incubator and acquired on a Guava EasyCyte system. The Guava MitoPotential Kit (catalog no. 4500-0250, Merck Millipore, Burlington, MA, USA) was used according to the manufacturer’s instructions. Values were calculated on the basis of the control group [23].

### 4.8. Live and Dead Assay

A total of 1 × 10^6^ cells were plated on six-well plates. The cells were treated with the GSK864 compound for 48 h. 7-AAD added together with The Guava MitoPotential Kit used for live dead assay. Plates were incubated for 30 min at 37 °C in a 5% CO_2_ incubator and acquired on a Guava EasyCyte system, the samples were immediately assessed by flow cytometry. Values were calculated on the basis of the control group [23].

### 4.9. Statistical Analysis

Differences between control and urolithin A- and urolithin B-treated groups were determined by one-way analysis of variance (ANOVA) for multiple groups using GraphPad Prism 8.0 (GraphPad Software, La Jolla, CA, USA). Test results with *p* < 0.05 were considered statistically significant.

## 5. Conclusions

In conclusion, we showed that the inhibition of wild-type IDH modulates the overall intercellular metabolism of leukemic cells. The inhibition of wild-type IDH1 facilitates oxidative stress by modulating pathways associated with the glutamine pathway, which ultimately results in apoptosis. Our results provide clues for identifying novel metabolic targets that are crucial for maintaining viability in wild-type IDH1 containing leukemic cells.

Limitation and future work: we will compare our results with in vivo and IDH1/2 knock-out models.

## Data Availability

The data presented in this study are available on request from the corresponding authors.

## Supplementary Materials

The following supporting information can be downloaded at: https://www.mdpi.com/article/10.3390/ijms23126653/s1.
