# Correction: Shait Mohammed et al. Profiling the Effect of Targeting Wild Isocitrate Dehydrogenase 1 (IDH1) on the Cellular Metabolome of Leukemic Cells. *Int. J. Mol. Sci.* 2022, *23*, 6653

**DOI:** 10.3390/ijms27146174

**Published:** 2026-07-10

**Authors:** Mohammed Razeeth Shait Mohammed, Faisal Alzahrani, Salman Hosawi, Hani Choudhry, Mohammad Imran Khan

**Affiliations:** 1 Department of Biochemistry, Faculty of Science, King Abdulaziz University, Jeddah 21589, Saudi Arabia; faahalzahrani@kau.edu.sa (F.A.); shosawi@kau.edu.sa (S.H.); hchoudhry@kau.edu.sa (H.C.); 2 Centre of Artificial Intelligence for Precision Medicines, King Abdulaziz University, Jeddah 21589, Saudi Arabia; 3 Embryonic Stem Cells Unit, King Fahd Medical Research Center, King Abdulaziz University, Jeddah 21589, Saudi Arabia

There were some errors in the original publication [1], due to the author’s negligence.

Some texts need to be updated in Sections 2.6, 4.7 and 4.8.

In Section 2.6, the sentence “Cytoxan Red was used to differentiate live” should be updated to the following version:

“7-Aminoactinomycin D (7AAD) staining was used to differentiate live”.

In Section 4.7, the sentence “1 μg/mL JC-1 Solution reagent was added. After one hour of incubation at 37 °C and 5% CO_2_, PI was added before 5 min and analyzed by flow cytometry.” should be updated to the following version:

“A volume of 2 μL 100X JC-1 solution and 2 μL of 7-AAD was then added to each well. Plates were incubated for 30 min at 37 °C in a 5% CO_2_ incubator and acquired on a Guava EasyCyte system. The Guava MitoPotential Kit (catalog no. 4500-0250, Merck Millipore, Burlington, MA, USA) was used according to the manufacturer’s instructions”.

In Section 4.8, the sentences “Cytoxan RED Solution reagent was added. After one hour of incubation at 37 °C at 5% CO_2_ and analysis,” should be updated to the following version:

“7-AAD added together with The Guava MitoPotential Kit used for live dead assay. Plates were incubated for 30 min at 37 °C in a 5% CO_2_ incubator and acquired on a Guava EasyCyte system,”.

The authors state that the scientific conclusions are unaffected. This correction was approved by the Academic Editor. The original publication has also been updated.
